# Identification of Vimentin as a Potential Therapeutic Target against HIV Infection

**DOI:** 10.3390/v8060098

**Published:** 2016-06-15

**Authors:** Celia Fernández-Ortega, Anna Ramírez, Dionne Casillas, Taimi Paneque, Raimundo Ubieta, Marta Dubed, Leonor Navea, Lila Castellanos-Serra, Carlos Duarte, Viviana Falcon, Osvaldo Reyes, Hilda Garay, Eladio Silva, Enrique Noa, Yassel Ramos, Vladimir Besada, Lázaro Betancourt

**Affiliations:** 1 Center for Genetic Engineering and Biotechnology, Ave 31/158 and 190, Cubanacán, Playa, P.O. Box 6162, Havana 10600, Cuba; anna.ramirez@cigb.edu.cu (A.R.); dionne.casillas@cigb.edu.cu (D.C.); taimi.paneque@cigb.edu.cu (T.P.); raimundo.ubieta@cigb.edu.cu (R.U.); lilacastellanos@infomed.sld.cu (L.C.-S.); carlos.duarte@cigb.edu.cu (C.D.); viviana.falcon@cigb.edu.cu (V.F.); osvaldo.reyes@cigb.edu.cu (O.R.); hilda.garay@cigb.edu.cu (H.G.); yassel.ramos@cigb.edu.cu (Y.R.); vladimir.besada@cigb.edu.cu (V.B.); lazaro.betancourt@cigb.edu.cu (L.B.); 2 Laboratory for AIDS Research, Carretera de Tapaste y Autopista Nacional, San José de las Lajas, Mayabeque CP 32700, Cuba; cicdc@infomed.sld.cu (M.D.); riverora@infomed.sld.cu (L.N.); eladio.silva@infomed.sld.cu (E.S.); enrnoa@infomed.sld.cu (E.N.)

**Keywords:** leukocyte extract, vimentin, intermediate filaments, cytoskeleton, HIV, anti-HIV activity, proteomics

## Abstract

A combination of antiviral drugs known as antiretroviral therapy (ART) has shown effectiveness against the human immunodeficiency virus (HIV). ART has markedly decreased mortality and morbidity among HIV-infected patients, having even reduced HIV transmission. However, an important current disadvantage, resistance development, remains to be solved. Hope is focused on developing drugs against cellular targets. This strategy is expected to prevent the emergence of viral resistance. In this study, using a comparative proteomic approach in MT4 cells treated with an anti-HIV leukocyte extract, we identified vimentin, a molecule forming intermediate filaments in the cell, as a possible target against HIV infection. We demonstrated a strong reduction of an HIV-1 based lentivirus expressing the enhanced green fluorescent protein (eGFP) in vimentin knockdown cells, and a noteworthy decrease of HIV-1 capsid protein antigen (CAp24) in those cells using a multiround infectivity assay. Electron micrographs showed changes in the structure of intermediate filaments when MT4 cells were treated with an anti-HIV leukocyte extract. Changes in the structure of intermediate filaments were also observed in vimentin knockdown MT4 cells. A synthetic peptide derived from a cytoskeleton protein showed potent inhibitory activity on HIV-1 infection, and low cytotoxicity. Our data suggest that vimentin can be a suitable target to inhibit HIV-1.

## 1. Introduction

Despite notable advances made during the last decade on antiretroviral therapies against the human immunodeficiency virus (HIV), acquired immunodeficiency syndrome (AIDS) continues to rank among the top 10 causes of death worldwide, and still represents the second most important cause of mortality in low-income countries [1]. The epidemic has continued to grow, although by 2012 the global number of new infections had declined by 33% as compared to 2001 [2]. In 2013, it was estimated that 35 million people were living with HIV, while 1.5 million died of AIDS-related causes [3].

Current treatment guidelines for HIV disease recommend the use of antiretroviral drug (ARVs) regimes that combine at least two, and preferably three, drugs from two or more drug classes to achieve viral suppression [4]. This combination is known as highly active antiretroviral therapy (HAART) or antiretroviral therapy (ART) [5,6]. ART has been effective because it controls viral replication and partially reconstitutes immune defenses, ultimately decreasing HIV-related morbidity and mortality [7,8,9]. However, the use of the ART is fundamentally limited by the development of viral resistance [10], and in addition, ART has been associated with bone, cardiovascular and cognitive disorders, does not eliminate viral reservoirs, and requires lifelong daily therapy [11,12].

New biological approaches to the treatment of HIV infection have been examined in an effort to overcome the disadvantages of current therapies. Since viruses are obligate intracellular parasites without an independent metabolism that rely on the host machinery for their replication [10], it has been proposed that targeting host proteins involved in the HIV replication cycle might represent a potentially rewarding research avenue for the development of novel ART alternatives. Practical evidence has now validated this therapeutic approach: maraviroc, an FDA-approved drug, binds to the CCR5 viral co-receptor and blocks its interaction with the viral envelope protein gp120, thus preventing viral entry [13,14]. Similarly, drug candidates targeting other viral receptors such as CXCR4 [15] and CD4 [16], are currently in an advanced stage of development. The use of host proteins as therapeutic targets might limit the emergence and selection of drug-resistant viral strains, as the drug would be acting on stable cellular components and not on highly variable viral molecules [10,17].

Several cytoskeleton proteins have been found to play a role on the HIV life cycle. The cytoskeleton is a dynamic structure composed of numerous proteins forming networks [18]. In mammalian cells, it consists of three filament systems: microtubules, microfilaments and intermediate filaments (IFs) [19]. Two of the most widely studied cytoskeleton proteins are actin, which plays an important role in HIV entry, and tubulin, involved in the cell-to-cell transmission of HIV virions [20,21,22]. Vimentin, a protein that forms intermediate filaments in cells of mesenchymal origin, has also been shown to interact with HIV proteins. *In vitro* studies have demonstrated that the HIV-1 protease (HIV-1 PR) cleaves human vimentin between Leu 422 and Arg 423. The microinjection of HIV-1 PR into human fibroblasts increased the percentage of cells with an abnormal distribution of vimentin intermediate filaments [19], and the N-terminal polypeptides generated through the cleavage of vimentin by HIV-1 PR are responsible for changes in the nuclear architecture of these cells [23]. Similar vimentin degradation patterns were observed in human oral gingival epithelial cell lysates from HIV-infected individuals [24]. The HIV viral infectivity factor (Vif) is found predominantly in the cytoplasm, where it colocalizes with vimentin. Reagents that affect the structure of vimentin filaments also affect the location of Vif. It has also been observed that the association of this viral protein with vimentin can collapse the intermediate filament network [25].

Our group has previously reported the presence of an anti-HIV activity in human dialyzable leukocyte extract (DLE). When DLE was subjected to gel filtration, a strong anti-HIV activity was identified in one chromatographic fraction, B1 [26,27]. In the present study, we use comparative proteomics to identify vimentin as one of the proteins modulated by this DLE fraction in the MT4 cell line. Furthermore, we demonstrate that modifying the levels of endogenous vimentin or the structure of vimentin IFs lead to the inhibition of HIV replication. Finally, we demonstrate that HIV replication can be inhibited *in vitro* with a synthetic peptide that targets vimentin.

## 2. Materials and Methods

### 2.1. Cell Cultures and Reagents

The MT4 cell line, obtained from the National Institute for Biological Standards and Control, UK, reference ARP016, was cultivated in RPMI 1640 medium (Hyclone, Logan, UT, USA) supplemented with 10% heat-inactivated fetal bovine serum (FBS, PAA, Ontario, Canada) and 0.05 mg/mL gentamicin (Sigma-Aldrich, St. Louis, MO, USA) under a humidified atmosphere of 5% CO_2_ at 37 °C. MT4sh/Vim and MT4mock cells were obtained as described below and cultivated as described for MT4 cells. The doubling times (DT) for MT4sh/Vim and MT4mock cells were determined. The cells were seeded at 21,000 per well and total viable cells were quantified every 24 h for 7 days by Trypan blue dye exclusion assay in Neubauer haemocytometer. The doubling time was calculated as DT = T × ln2/ln(Xe/Xb), where T is the incubation time, Xb is the cell number at the beginning of the incubation time and Xe is the cell number at the end of the incubation time. CIGB-210 is a 25-mer peptide derived from human keratin 10 whose sequence is RVTQMNLNDRLASLYDKV. Penetrating peptide (PP) is a peptide that contains the HIV-1 Tat cell penetrating peptide whose sequence is GRKKRRQRRRPPQACWMSPRHLGTC [28]. Carboxyfluorescein and biotin labelled peptides were obtained coupling a carboxyfluorescein or biotin molecule to the N-terminal residue through the formation of an amide bond during peptide synthesis. Peptide identities were confirmed by electrospray ionization mass spectrometry (ESI-MS, Waters, Milford, MA, USA). Peptides were synthesized at the Peptide Synthesis Department of the Center for Genetic Engineering and Biotechnology in Havana, Cuba. Fraction B1 was obtained from a human leukocyte extract from healthy donors by gel filtration chromatography on Sephadex G-15 (Pharmacia Biotech, Piscataway, NJ, USA) as described [26]. Once collected, the fraction was lyophilized and stored at −20 °C for later use.

### 2.2. Comparative Proteomics

The MT4 cell line was treated with a leukocyte extract exhibiting anti-HIV activity (B1 fraction) for 3 and 24 h, and the resulting protein expression profile was compared to that of untreated cells using a Two Dimensional Electrophoresis/Mass Spectrometry approach (2DE/MS). Cells were disrupted in a lysis solution containing 7 M urea, 2 M thiourea, 2% 3-[(3-cholamidopropyl)dimethylammonio]-1-propanesulfonate (CHAPS), 0.5% 3-[*N*,*N*-Dimethyl(3-myristoylaminopropyl)ammonio]propanesulfonate (ASB-14), 15% glycerol, 2% dithiothreitol (DTT), and protease inhibitors, and the resulting samples were centrifuged at 12,000 rpm for 20 min. The supernatant was collected and the pellet was submitted to a second lysis procedure. The second supernatant was pooled with the first one, the preparation was further incubated for 1 h at 37 °C to reduce all disulfide bonds, and cysteines were blocked with 5% acrylamide. The samples were then delipidated with ethyl alcohol, and DNA was removed by centrifugation at 60,000× *g* for 3 h. Triplicate two-dimensional gel electrophoreses for each sample were carried out on immobilized pH gradient (IPG) strips 4–7 for isoelectric focusing and 12.5% Tris-Tricine polyacrylamide gels for the second dimension. Proteins were detected by silver staining and gel images were analyzed with Melanie 5 software (GeneBio, Geneva, Switzerland). Normalized spot volumes were used for relative quantitation, and the spots to be used for identification were cut out from the gels and digested with trypsin. The obtained proteolytic peptides were analyzed by mass spectrometry on a hybrid type QTOF-2 instrument equipped with a nanospray ionization source. The most intense signals on the resulting electrospray ionization mass spectra were sequenced by collision-induced dissociation. For protein identification, the ESI-MS/MS spectra were submitted to the MS/MS ion search option of the MASCOT program (v 2.3.02, Matrix Science, London, UK), setting propionamide-cysteine as a fixed modification and the deamidation of glutamine and asparagine as well as methionine sulfoxide as variable modifications. Other restrictions to MASCOT were an *m*/*z* tolerance of 0.3 Da for precursor and fragment ions, enzyme digestion with trypsin, and up to one missed cleavage. Queries were carried out by searching the non-redundant protein sequence database of the National Center for Biotechnology Information (NCBI, Bethesda, MA, USA).

### 2.3. Western Blotting to Detect Vimentin

Cellular extracts were separated by electrophoresis on 10% polyacrylamide gels and transferred to Hybond-P polyvinylidene difluoride membranes (Sigma-Aldrich) that were blocked afterwards with 5% nonfat dried milk (OXOID, Hampshire, United Kingdom) in phosphate buffer saline (PBS) for 1 h. Monoclonal antibody V9, specific for human vimentin (Sigma-Aldrich), was used for identification at 6.8 µg/mL. Anti-β-actin (Sigma-Aldrich) (3.1 µg/mL) or anti-G3PDH (1 µg/mL) antibodies were used as loading controls. All antibodies were diluted in PBS containing 5% nonfat dried milk and incubated for 1.5 h. A horseradish peroxidase (HRP)-conjugated anti-mouse IgG antibody (Sigma-Aldrich) at 8.8 µg/mL was used as secondary antibody. The activity of the HRP enzyme was visualized after 1 min incubation with 0.5 mg/mL diaminobenzidine (Sigma-Aldrich) in PBS, in the presence of 0.015% hydrogen peroxide (Merck, Darmstadt, Germany). Quantification of bands from Western blots was done using Image J software.

### 2.4. Construction of the Expression Transfer Plasmid Encoding Short Hairpin RNA Targeting Vimentin and the Mock Transfer Plasmid

Two oligonucleotides containing the sense and antisense sequences of a small interfering RNA (siRNA) spaced by a loop were designed and synthesized from the coding sequence of the vimentin gene to construct a short hairpin RNA (shRNA) targeting vimentin: (5′ ATCAACACCGAGTTCAAGACTCGAGTTGAACTCGGTGTTGATGGTTTTTT 3′ and 5′ TCGAAAAAAACCATCAACACCGAGTTCAACTCGAGTCTTGAACTCGGTGTTGATGGCC 3′). The oligonucleotides were phosphorylated, annealed and ligated downstream to the mouse U6 promoter in the pBSM-U6 plasmid (derived from pBluescript SK, Stratagene, La Jolla, CA, USA). Afterwards, the region containing the promoter, and the shRNA targeting vimentin, was transferred to the pLenti6/V5-GW/lacZ vector (Invitrogen, Waltham, MA, USA) at the Cla I and Sac II sites (Supplementary Materials, Figure S1). The lentiviral mock transfer vector was the pLenti6-GW/LacZ plasmid devoid of the U6 promoter and the shRNA coding sequence.

### 2.5. Construction of the Transfer Plasmid Encoding the eGFP Reporter Gene

The plasmid was provided by the Animal Biotechnology Department of the Center for Genetic Engineering and Biotechnology. It was originated from the pLenti6/V5-GW/lacZ vector. The plasmid contains the enhanced green fluorescent protein (eGFP) sequence under simian virus 40 (SV40) promoter. This vector also includes a central polypurine tract, two multiple cloning sites, and the posttranscriptional regulatory element of woodchuck hepatitis virus.

### 2.6. Production of Lentiviral Vector Particles

Lentiviral vector particles pLenti-shRNAvim, mock lentiviral vector or pLWG were packed in the 293T cell line transduced with four plasmids. These plasmids were pLP1, pLP2, pLP/VSVG and either the expression transfer plasmid encoding shRNA targeting vimentin, or the mock transfer plasmid, or the transfer plasmid encoding eGFP. The pLP1 vector encodes for gag/pol polyproteins of HIV-1. The pLP2 plasmid bears the sequence encoding for the HIV-1 Rev protein. The pLP/VSVG encodes for the surface protein of the vesicular stomatitis virus (VSV-G). The expression transfer plasmid encoding shRNA and targeting vimentin, the mock transfer plasmid, and the transfer plasmid encoding eGFP contain the genome of their respective lentiviral vector particles. All plasmids were amplified in the Escherichia coli XL-1 strain growing under ampicillin selection. The four plasmid vectors were transfection-quality purified by column chromatography and put together into contact with the 293T packaging cell line in the presence of 0.81 mg/mL polyethyleneimine 25 kDa, pH 7. The cells were incubated for 48 h at 37 °C under a humidified atmosphere of 5% CO_2_, and virions were further purified by ultracentrifugation at 20,000× *g*.

### 2.7. Generation of the Vimentin Stable Knockdown Cell Line (MT4sh/Vim) and the MT4mock Cell Line

The MT4 cell line was transduced with the pLenti-shRNAvim or the mock lentiviral vectors. Recombinant cells were selected for blasticidin resistance, cloned by a limiting dilution assay and cultivated in an RPMI medium supplemented with 10% FBS under a humidified atmosphere of 5% CO_2_ at 37 °C. Total proteins were extracted from the cultures and the silencing of vimentin was demonstrated by Western blotting.

### 2.8. Early Steps of the HIV-1 Replication Assay

The MT4sh/Vim and MT4mock cell lines were transduced with a lentiviral vector bearing part of the HIV-1 genome, lacking the genes involved in infectivity and entry (pLGW). The expression of eGFP was used as a viral cycle indicator until replication. Results were followed by fluorescence microscopy (Olympus, Tokio, Japan) and samples were analyzed by fluorescence-activated cell sorting (FACS) Partec Pas III (Partec, Muenster, Germany).

### 2.9. HIV-1 Replication Assay

MT4 cells and the vimentin knockdown cell line (MT4sh/Vim) were cultured in RPMI medium supplemented with 10% FBS under a humidified atmosphere of 5% CO_2_ at 37 °C. They were challenged with the BRU viral strain of HIV at multiplicities of infection (m.o.i.) of 0.1, 0.01 or 0.001, and viral replication was followed by determining the concentration of HIV-1 capsid protein antigen (CAp24) in culture supernatants after 96, 120 or 168 h by enzyme-linked immunosorbent assay (ELISA). The results were expressed as HIV-1 inhibition percentages, calculated as I = (p24U − p24T/p24U) × 100, where p24U and p24T represent CAp24 concentration in untreated cells and treated cells, respectively.

### 2.10. Cytotoxicity Assay

Cellular cytotoxicity was evaluated by the Trypan blue dye exclusion assay. A total of 5 × 10^5^ cells were seeded into 24-well plates and treated or not with different doses of CIGB-210 for 24 or 144 h at 37 °C under a humidified atmosphere of 5% CO_2_. Afterwards, the cultures were homogenized and a sample from each one was stained with 0.4% Trypan blue (Sigma-Aldrich, USA) and counted in a Neubauer haemocytometer under an optical microscope (Olympus, Japan). The assays were performed in triplicate, and the results were reported as % viability, mean ± standard deviation.

### 2.11. Transmission Electron Microscopy

MT4 and MT4sh/Vim cell lines were fixed in 3.2% glutaraldehyde for 1 h at 4 °C and then fixed in 2% osmium tetroxide for 1 h at 4 °C. They were subsequently washed with 0.1 M PBS, pH 7.2, and dehydrated at increasing ethanol concentrations (30%, 50%, 70% and 100%) for 10 min each at 4 °C. Inclusion was carried out and ultrathin 40–50 nm width sections were prepared with an ultramicrotome (LKB, Uppsala, Sweden), which were placed on 400 holes nickel trays. After staining saturated uranyl acetate and lead citrate, the sections were examined under a JEOL JEM-1400 electron microscope (JEOL, Tokio, Japan). Five nickel trays were analyzed at different magnifications. Fifteen microphotographs were taken for each tray.

### 2.12. Immunofluorescence Analysis

The MT4sh/Vim, MT4mock and MT4 cell lines were attached to poly-l-lysine coated slides (Sigma-Aldrich, USA) for 30 min. The slides were washed with PBS and fixed by immersion for 10 min at −20 °C in acetone-methanol solution (*v*/*v*). The slides were dried at room temperature, washed with PBS and blocked for 30 min a 37 °C with 1% bovine serum albumin (BSA) in PBS. The slides were incubated with anti-vimentin monoclonal antibody V9 (Sigma, USA) at 4.5 µg/mL for 1 h at room temperature. The slides were washed three times with PBS for 5 min with gentle agitation and then incubated with a FITC conjugated anti-mouse IgG antibody at 50 µg/mL for 1 h at room temperature. The slides were washed three times with PBS, treated for 30 s with propidium iodine (Sigma-Aldrich, USA) at 10 µg/mL and washed again three times as described before. Then 10 µL of the VECTASHIELD mounting medium (VECTOR, Burlingame, CA, USA) were added to the coverlids and the cells were covered. The slides were sealed with regular transparent nail varnish and the fluorescence was visualized in a BX51 fluorescence microscopy (OLYMPUS, Japan).

### 2.13. Cell Penetration Assays

CIGB-210 uptake by flow cytometry: A total of 5 × 10^4^ MT4 cells were seeded into 96-well plates and treated or not with different doses of fluorescein labelled CIGB-210 for 15 min, 60 min or 24 h at 37 °C under a humidified atmosphere of 5% CO_2_. Carboxyfluorescein labelled PP was used as positive control of penetration. MT4 cells were collected and centrifuged at 300× *g* for 10 min. They were washed twice with PBS. To discriminate between attached and internalized peptides, a fluorescein quenching Trypan blue step was introduced as described elsewhere [29,30]. Cells were incubated for 5 min with a 0.4% (*w*/*v*) Trypan blue solution (Sigma-Aldrich, USA), washed twice with PBS by centrifugation and resuspended in 1.5 mL of PBS for flow cytometry analysis using a PAS III flow cytometer (Partec, Muenster, Germany). Data were processed with the WinMDI 2.8 software.

CIGB-210 uptake by immunofluorescent microscopy: MT4 cells were incubated with 10 µM of biotinylated CIGB-210 for 24 h. Cells treated with biotin labelled PP were used as positive control of penetration. Cells were harvested, washed with PBS by centrifugation at 250× *g* for 5 min and attached to poly-l-lysine coated slides (Sigma-Aldrich, USA) for 30 min. The samples were then fixed with a 4% paraformaldehyde solution for 1.5 h at 4 °C. PBS-5% Tween 20 was added over 10 min. They were blocked for 30 min at 37 °C with a 1% BSA solution in PBS. Cells were incubated for 1 h at 37 °C with streptavidin-FITC (Dako, Glostrup, Denmark). A final incubation step with 10 ug/mL propidium iodine (Sigma-Aldrich, USA) over 30 s was implemented for nuclear staining. Then, 10 µL of the VECTASHIELD mounting medium (VECTOR, Burlingame, CA, USA) were added to the coverlids and the cells were covered. The slides were sealed with regular transparent nail varnish and the fluorescence was visualized in a BX51 fluorescence microscopy (OLYMPUS, Japan).

### 2.14. Statistical Analysis

Statistical processing was performed using GraphPad Prism version 6.01 (GraphPad Software, La Jolla, CA, USA).

## 3. Results

### 3.1. HIV-1 Inhibition and Downmodulation of Vimentin in MT4 Cells Treated with an Anti-HIV Fraction

**Figure 1:**
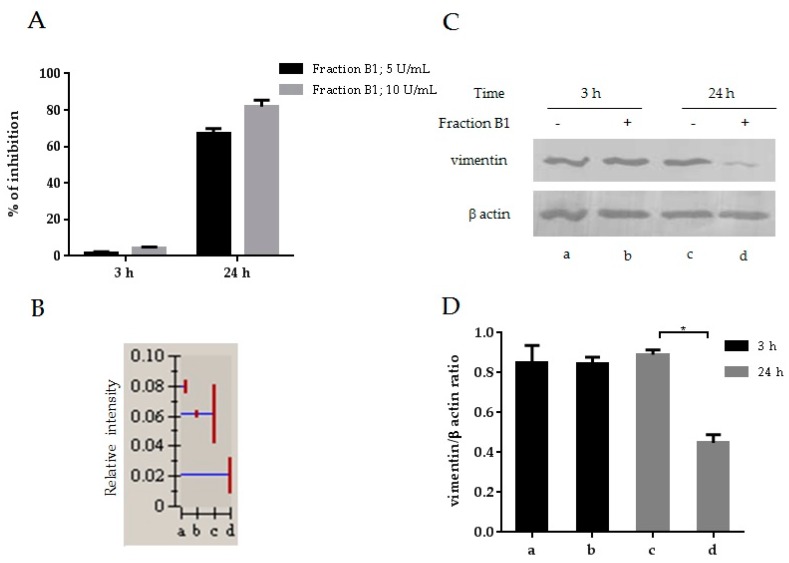
HIV-1 inhibition and downmodulation of vimentin in MT4 cells treated with the B1 fraction. (**A**) MT4 cells were treated with 5 or 10 U/mL of fraction B1 for 3 or 24 h before the BRU viral strain challenge (m.o.i. = 0.1). CAp24 was measured in culture supernatants seven days after infection; (**B**) Relative vimentin protein expression of untreated and B1 treated MT4 cells were obtained from a comparative proteomic experiment. Blue line: relative intensity; red line: standard deviation; (**C**) Western blot analysis of vimentin expression in untreated and treated MT4 cells with the B1 fraction, β-actin served as a loading control; (**D**) Band intensities were quantified and the ratio vimentin/β-actin was calculated. Error bars mean standard deviation. Mann–Whitney test was applied for calculation of significance; * *p* < 0.05. Data are representative from three experiments. a: MT4 cells untreated, 3 h; b: MT4 cells treated with the B1 fraction for 3 h; c: MT4 cells untreated, 24 h; and d: MT4 cells treated with the B1 fraction for 24 h.

Our group has previously reported a remarkable anti-HIV activity in MT4 cells pre-treated with a human dialyzable leukocyte extract (DLE) for 24 h or more. In contrast, no anti-HIV activity was observed when MT4 cells were treated for only 3 h [26,27]. A chromatographic fraction, B1, obtained from DLE by gel filtration, showed a potent anti-HIV activity when cells were treated for 24 h (Figure 1A). CAp24 antigen was measured seven days post infection. The treatment with the B1 fraction showed no cytotoxic effect in MT4 cells (Supplementary Materials, Figure S2). MT4 cells were treated for 3 or 24 h with B1 and a comparative proteomic analysis was performed. The resulting protein expression profile was compared to a control of untreated cells cultured for 3 or 24 h, respectively. Gel images were analyzed by the Melanie 5 software and proteins were identified using mass spectrometry. A decrease in vimentin protein expression was detected in MT4 cells treated with B1, especially after 24 h of treatment (Figure 1B). Downmodulation of vimentin was confirmed by Western blot analysis of MT4 cells treated with the chromatographic B1 fraction after 24 h (Figure 1C). Quantification of bands from Western blots was done using Image J software (Figure 1D).

### 3.2. Generation of Vimentin Stable Knockdown and Mock Cell Lines

**Figure 2:**
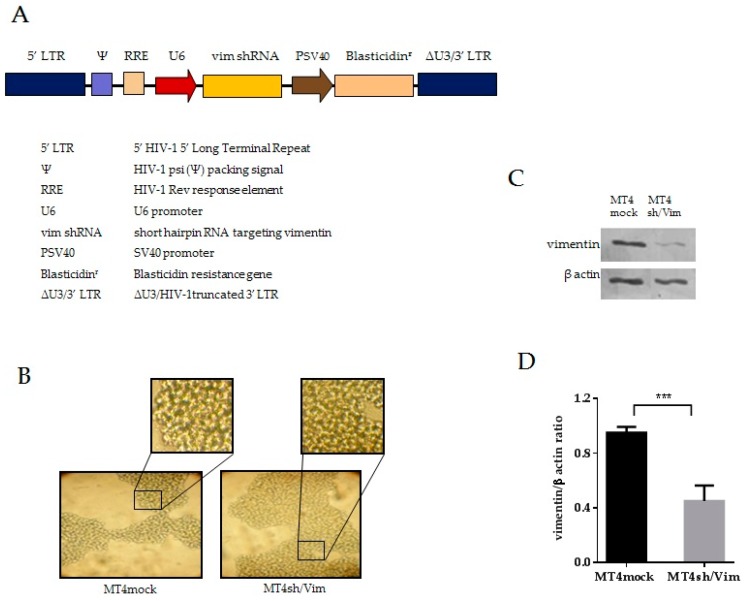
Generation of vimentin stable knockdown cell line. (**A**) Lentiviral vector genome encoding a short hairpin RNA targeting vimentin. Lentivirus was produced by transfecting the packing cell line 293FT with four plasmids providing the RNA genome and the viral proteins required for constructing the lentiviral particles; (**B**) Optical microscopy images of vimentin knockdown (MT4sh/Vim) and control (MT4mock) cell lines at 20×; (**C**) Western blot analysis of vimentin expression levels performed with protein extracts of MT4sh/Vim and MT4mock cell lines. β-actin served as a loading control; (**D**) Band intensities were quantified using Image J software and the ratio vimentin/β-actin was calculated. Graphs represent averages of quantifications and error bars mean standard deviation. Unpaired *t* test with Welch’s correction was applied for calculation of significance; *** *p* < 0.05. Data are representative from three experiments.

A lentiviral vector encoding a shRNA targeting vimentin was used to induce vimentin knockdown in the MT4 cell line. An expression cassette constructed for human U6 promoter-driven expression of short hairpin RNA with exact homology to the human vimentin mRNA was cloned in the HIV-1 based lentiviral vector. MT4 cells were transduced with the shRNA containing the lentivirus (pLenti-shRNAvim) (Figure 2A) or the mock lentiviral vector (the lentivirus had neither the shRNA expression cassette nor the human U6 promoter). Individual clones were obtained by limiting dilution and vimentin knockdown monoclonal cell lines were cultured. A monoclonal cell line with a significant decrease in vimentin was selected and designated MT4sh/Vim. The cell line transduced with the mock lentiviral vector was designed as MT4mock cell line. The morphologic characteristics of both cell lines were similar (Figure 2B). Doubling times were of 35.2 and 34.6 h for MT4sh/Vim and MT4mock, respectively. Western blot analysis was performed to evaluate vimentin expression in both cell lines. A noteworthy reduction in vimentin expression levels was observed in MT4sh/Vim cells as shown in Figure 2C. Quantification of bands from Western blots was done using Image J software (Figure 2D).

### 3.3. Effect of Vimentin Knockdown on Early Steps of HIV-1 Replication

**Figure 3:**
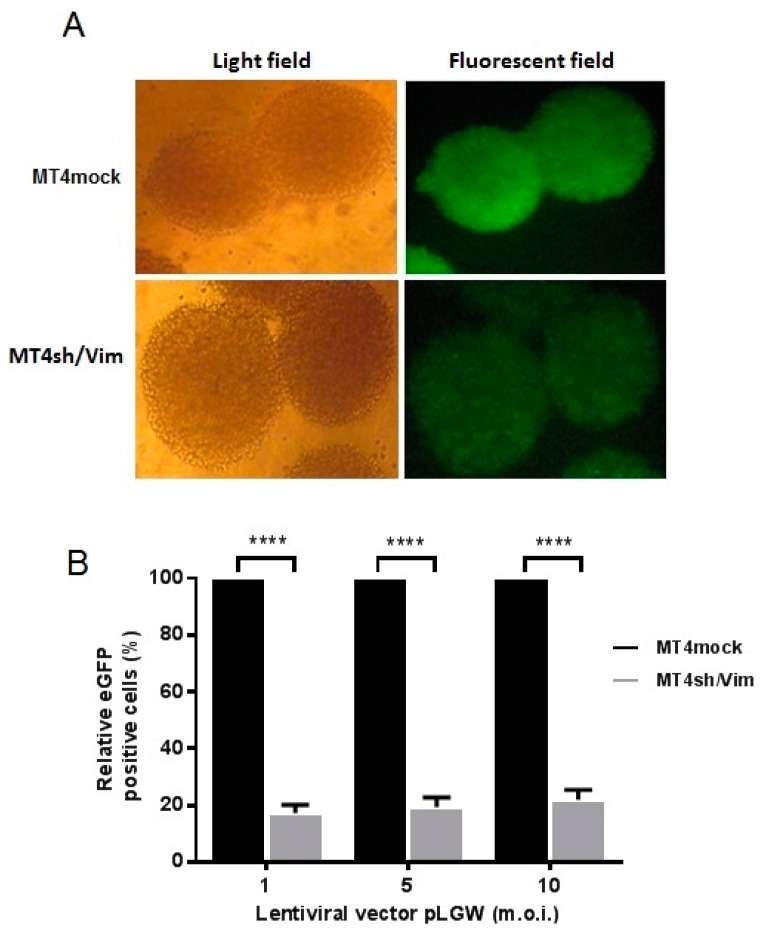
Vimentin silencing reduces the expression of an HIV-1 based lentiviral vector. (**A**) Fluorescence microscopy of transduced MT4sh/Vim and MT4mock cell lines with the enhanced green fluorescent protein (eGFP) expressing the lentiviral vector (pLGW). Cells were transduced at different m.o.i. and the eGFP expression was evaluated after 72 h. Images from fluorescence microscopy of transduced cell lines with the median concentration of lentivirus (m.o.i. = 5) are shown in light field and fluorescent field. 10 × magnification; (**B**) Relative percentage of eGFP positive cells measured by flow cytometry in MT4sh/Vim and MT4mock cells transduced with the eGFP expressing lentiviral vector based on HIV-1. The percentage of positive cells in the MT4mock transduced cells were considered 100%. Error bars mean standard deviation. Data are representative of three experiments. Bonferroni multiple comparisons test was done for statistical analysis. Vimentin silencing causes a significant decrease in the percentage of eGFP expressing cells, indicating inhibition in early pLGW lentivirus replication (**** *p* < 0.0001).

To determine whether the downregulation of vimentin confer defects in infectivity, the vimentin stable knockdown cell line, MT4sh/Vim, or MT4mock were challenged with a lentiviral vector based on HIV-1 (pLGW). This VSV-G pseudotyped lentivirus contains the eGFP gene as reporter. The 293FT packing cell line was transfected with plasmids providing *gag*, *pol* and *rev* HIV genes to produce lentiviral particles. Once the lentivirus enters the target cell the viral RNA is reverse-transcribed, then actively imported into the nucleus, stably integrated into the host genome and the eGFP is produced. eGFP expression was followed as a marker of successful integration of the virus. MT4sh/Vim and MT4mock cell lines were transduced with the HIV-1 lentiviral vector (pLGW) at different m.o.i. After 72 h, the eGFP expression was analyzed by FACS and fluorescence microscopy (Figure 3). A strong reduction in eGFP expression was observed in the vimentin knockdown cell line MT4sh/Vim compared to the intense fluorescence observed in the MT4mock cell line (Figure 3A). The light field images were included for a detailed observation of the cell clusters characteristics of MT4 cells and location. The quantitative determination by FACS of the number of eGFP expressing cells confirmed this result. Reduction percentages of eGFP positive cells were 83%, 81.5% and 78% at m.o.i. of 1, 5 and 10, respectively (Figure 3B). The evidence suggests that vimentin could be involved in the early steps of HIV-1 replication.

### 3.4. Inhibition of Replication Competent HIV-1 in Vimentin Knockdown Cell Line

**Figure 4:**
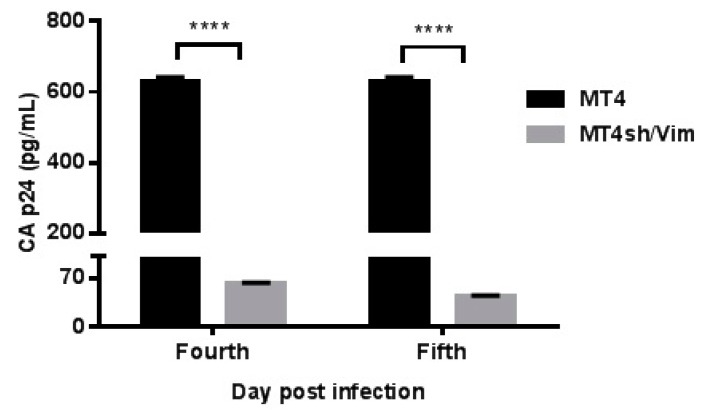
Inhibition of replication of competent HIV-1 in a vimentin shRNA-silenced cell line. MT4 vimentin knockdown cell line (MT4sh/Vim) and MT4 were infected with the HIV-1 BRU viral strain at a m.o.i. of 0.001. Virus was removed 1 h after viral challenge and CAp24 antigen was measured by enzyme-linked immunosorbent assay (ELISA) at days 4 and 5 after infection. Samples were run in triplicate and the experiment was repeated three times. Data represent the mean ± standard deviation of one representative experiment. Bonferroni multiple comparisons test was applied to compare HIV-1 replication between vimentin knockdown cell line and MT4 (**** *p* < 0.0001).

MT4sh/Vim cells were infected with a replication competent HIV-1BRU strain at an m.o.i. of 0.01. CAp24 was measured at four and five days post-infection in the culture supernatants. A significant reduction of CAp24 levels (90% and 93% inhibition as compared to MT4 cells) was observed in the vimentin knockdown cell line (Figure 4). Cell viability was similar at the beginning, during the assays and at their end. These results provide robust evidence that down-modulation of vimentin affects HIV-1 replication and suggest a role for vimentin in the HIV-1 replication cycle.

### 3.5. Structural Changes of Intermediate Filaments in MT4 Cells

**Figure 5:**
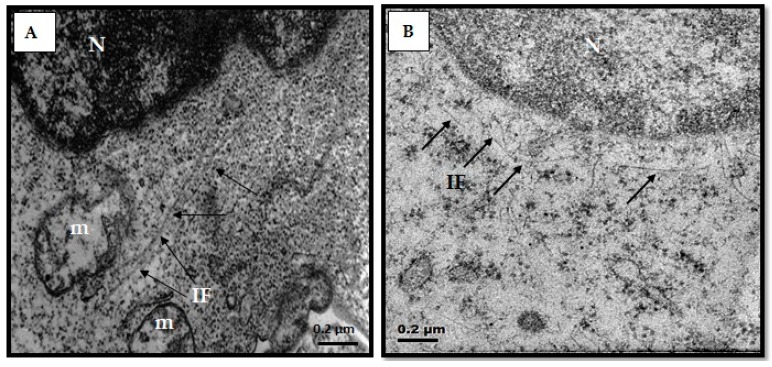

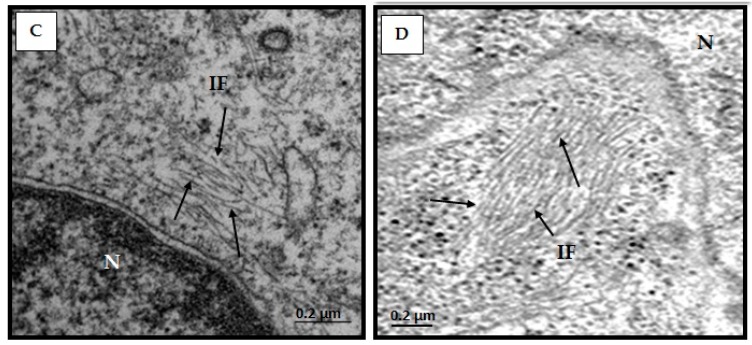
Intermediate filament structure in different cellular contexts. (**A**) Electron micrographs of the MT4 cell line. Large intermediate filaments can be observed; (**B**) MT4 cell line treated with the B1 fraction; (**C**,**D**) MT4 vimentin knockdown cells (MT4sh/Vim); (**B**–**D**) Modified intermediate filaments seemingly shorter as compared to the length of the filaments in MT4 cells. Arrows IF: Intermediate filaments, N: Nucleus, m: mitochondria. Bar = 0.2 µm.

Due to vimentin assembly in IFs we examined IFs structure using transmission electron microscopy in different cellular contexts: MT4 cells, MT4 cells treated with the B1 fraction and MT4sh/Vim cell line. IFs were distinguished as large filaments close to the cell nucleus in MT4 cells (Figure 5A). However, in MT4 cells treated with the B1 fraction, IFs appear to be shorter than in MT4 cells (Figure 5B). In vimentin knockdown cells, MT4sh/Vim, IFs were also apparently shorter than in MT4 cells (Figure 5C). Moreover, clusters of IFs were observed (Figure 5D). Structural changes of IFs were noted in both MT4 cells treated with the chromatographic fraction showing anti-HIV activity and in vimentin silenced MT4 cells. In both cellular contexts, HIV replication was impaired. The diameters of the filaments were measured on micrographs from cells in different experimental contexts and they ranged between 9 and 10 nm.

**Figure 6:**
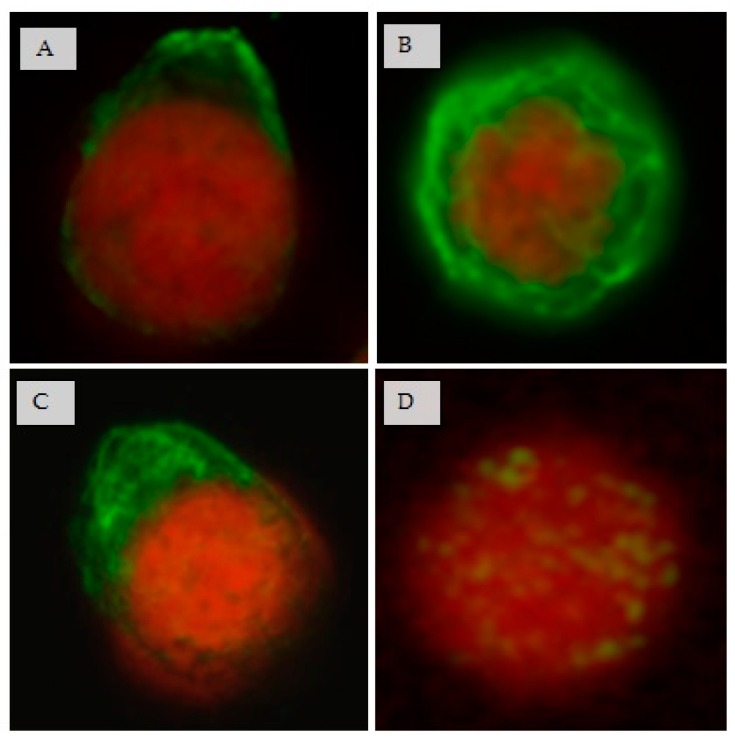
Immunofluorescence microphotographs of vimentin IFs in different cellular contexts: (**A**) MT4 cells; (**B**) MT4 cells treated with the B1 fraction; (**C**) MT4mock cells; and (**D**) MT4sh/Vim cells. Cells were fixed and vimentin IFs were visualized with an anti-vimentin mouse monoclonal antibody followed by a FITC conjugated anti-mouse IgG antibody (**green**). The nucleus was stained with propidium iodine (**red**). The localization of vimentin IFs in (**A**) and (**C**) was mostly delimited to one edge of the cell (**A**) 264 cells out of 291 for a 90.7%; and (**C**) 260 cells out of 323 for an 80.4%). In contrast, a redistribution in vimentin IFs was observed in (**B**) (226 cells out of 311 for a 72%). Scarce, scattered and unpolarized vimentin filaments were observed in MT4sh/Vim cells (**D**) 190 cells out of 207 for a 91.7%). 40× magnification.

The architecture of IFs was also examined by fluorescence microscopy. Vimentin IFs were observed polarized and generally delimited to one edge of the cell in MT4 and MT4mock cells (Figure 6A,C). In contrast, in MT4 cells treated with B1, vimentin IFs were redistributed around the cell nucleus and they seemed less compact and forming a network (Figure 6B). In MT4sh/Vim cells, vimentin IFs were also disperse through the cytoplasm and surrounding the cell nucleus (Figure 6D). Structural changes in vimentin IFs were observed in both MT4 cells treated with the chromatographic fraction showing anti-HIV activity and vimentin silenced MT4 cells. These results led to the idea that by modulating vimentin IFs structure it could be possible to inhibit HIV replication.

### 3.6. HIV-1 Inhibition by a Synthetic Peptide

Peptides from the 1A region of the central domain of vimentin and keratin IFs have been reported to disassemble *in vitro* preformed vimentin IFs. Microinjection of these peptides into hamster or mouse fibroblast cell lines changed the normal IF pattern of cells [31]. We evaluated the anti-HIV-1 activity of a keratin-10 1A-region derived peptide (CIGB-210), in a HIV-1 multi-round assay using the BRU wild type virus. MT4 cells were pre-treated with the peptide 24 h before viral challenge. Cells were infected at an m.o.i. of 0.001 over 1 h and a post-infection treatment with peptide was done at the same pre-treatment concentrations. Viral CAp24 antigen was measured by ELISA in the culture supernatants. An important HIV-1 inhibition was observed in the presence of different concentrations of CIGB-210. No cytotoxic effects were observed after six days of MT4 treatment with CIGB-210.

**Figure 7:**
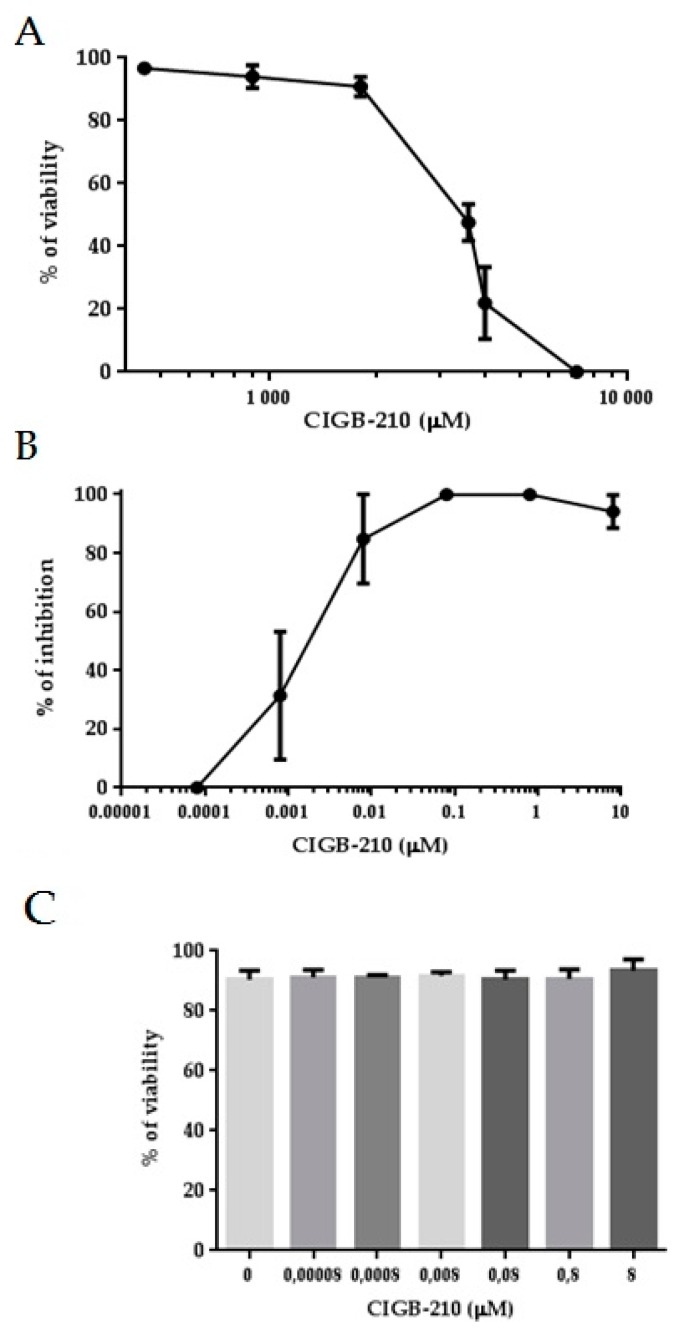
Cytotoxicity and anti-HIV activity of CIGB-210 in MT4 cell line. (**A**) MT4 cells were incubated with different concentrations of CIGB-210 for 24 h. Cell viability was assayed by the Trypan Blue dye exclusion method. Results were expressed as the percentage of viable cells. Samples were run in triplicate and the experiment was repeated three times. Data represent the mean ± standard deviation of one representative experiment. A CC50 of 1702 ± 255 µM was calculated using the CalcuSyn software; (**B**) MT4 cells were treated with different concentrations of CIGB-210 for 24 h, prior to HIV-1BRU infection (m.o.i. = 0.001). CIGB-210 was added again after infection and it was maintained until CAp24 antigen determination, five days post infection. Results are expressed as percentage of inhibition of HIV-1. Samples were run in triplicate. The data shown are average values ± standard deviation for three experiments. IC50 of 7.92 ± 1.76 nM was calculated using CalcuSyn software; (**C**) MT4 cells viability assessed by Trypan Blue dye exclusion method after treatment with different concentrations of CIGB-210 for 144 h. Data represent the mean ± standard deviation of two experiments performed in triplicate. Kruskal–Wallis test was applied for calculation of significance; *p* > 0.05. The treatment with CIGB-210 showed no cytotoxic effect in MT4 cells.

The half maximal inhibitory dose (IC50) and the half maximal cytotoxic concentration (CC50) of CIGB-210 were calculated using CalcuSyn software (Figure 7). CIGB-210 shows an IC50 of 7.92 ± 1.76 nM and a CC50 of 1702 ± 255 µM.

### 3.7. Effect of CIGB-210 on Intermediate Filaments in MT4 Cells

**Figure 8:**
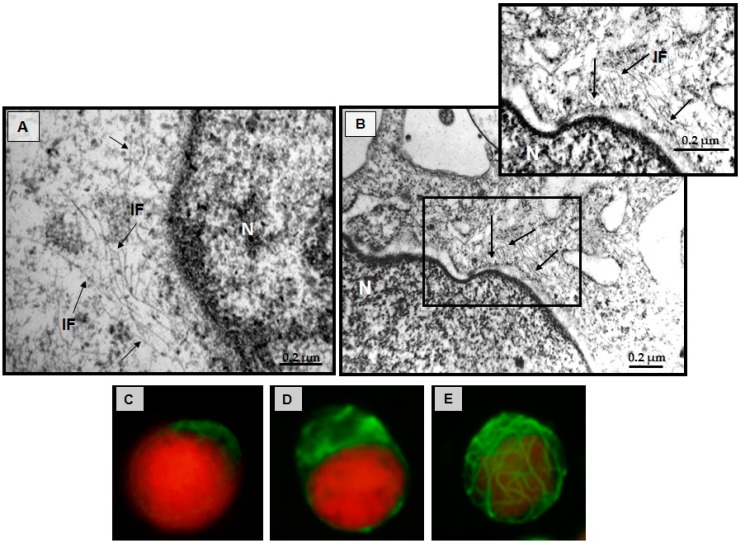
Visualization of vimentin IFs in MT4 cells treated with CIGB-210: (**A**) Electron micrographs of MT4 cells cultured for 24 h; (**B**) The MT4 cell line was treated with CIGB-210 at 200 µM for 24 h; and (**C**,**D**) Fluorescence micrographs of MT4 cells cultured for 24 h. The cells were fixed and vimentin IFs were tagged with an anti-vimentin monoclonal antibody followed by a FITC conjugated anti-mouse IgG antibody; (**E**) Fluorescence micrographs of MT4 cells treated with CIGB-210 at 40 µM for 24 h. Arrows IF: Intermediate filaments, N: Nucleus. Bar = 0.2 µm, Red: Cell nucleus, Green: Vimentin.

MT4 cells treated with CIGB-210 for 24 h were analyzed by transmission electron microscopy. Microphotographs show large intermediated filaments, typical of MT4 cells (Figure 8A). However, in MT4 cells treated with CIGB-210 IFs looked apparently shorter as shown in Figure 8B. The effect of CIGB-210 on the vimentin IFs was also assessed by fluorescence microscopy. Vimentin IFs were observed polarized and mostly restricted to one edge of the cell, and to a lesser extent around the nucleus (Figure 8C,D). In contrast, when MT4 cells were treated for 24 h with CIGB-210 at 40 µM (Figure 8E) the vimentin IFs were redistributed throughout the cytoplasm forming a network around the cell nucleus. No damage on the integrity of the cells was observed after treatment with CIGB-210. The diameters of the filaments were measured on micrographs from CIGB-210 treated and untreated MT4 cells and they ranged between 9 and 10 nm.

### 3.8. Cell Penetration of CIGB-210

**Figure 9:**
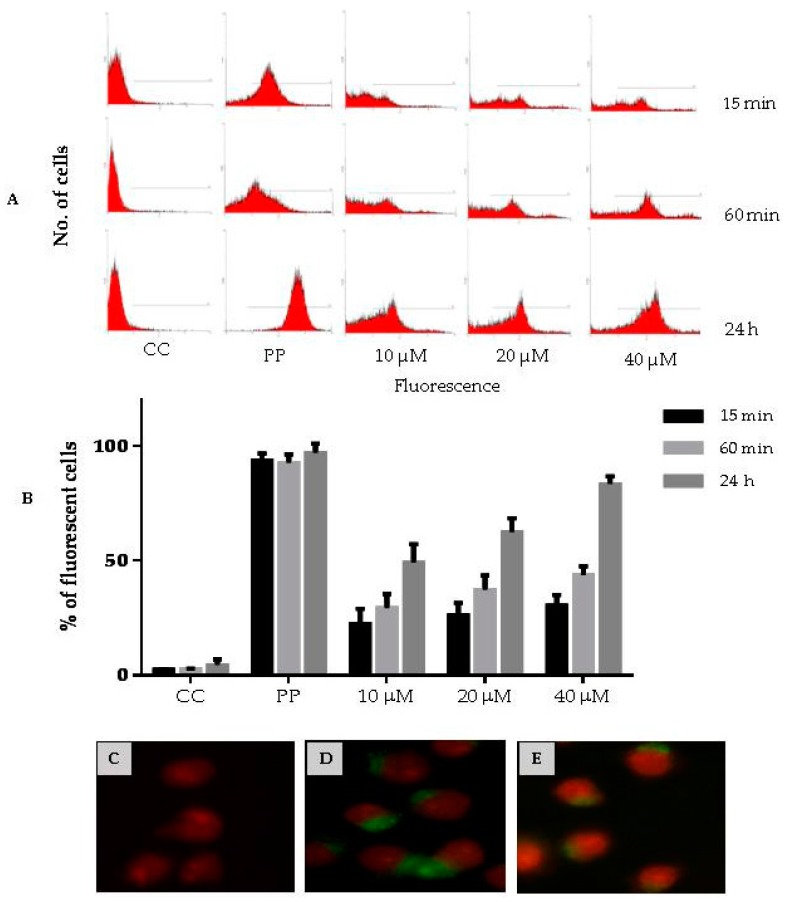
Penetration of CIGB-210 into the MT4 cell line. (**A**,**B**) CIGB-210 uptake assessed by flow cytometry. MT4 cells were incubated with 10, 20 or 40 µM of carboxyfluorescein labeled CIGB-210 for 15 min, 60 min or 24 h. External fluorescence was quenched by addition of 0.4% Trypan blue. CC: Control untreated MT4 cells; PP: carboxyfluorescein labeled peptide containing the Tat cell penetrating peptide used as positive control; (**A**) Flow cytometry histograms; and (**B**) percentage of fluorescent cells for each experimental condition. Data represent the mean ± standard deviation of three experiments performed in triplicate; (**C**–**E**) CIGB-210 uptake assessed by fluorescence microscopy. MT4 cells were treated with: 10 µM biotinylated CIGB-210 (**E**); 10 µM biotinylated Tat cell penetrating peptide (**D**); or medium (**C**) for 24 h, fixed and visualized with a streptavidin-FITC conjugate. The nucleus was stained with propidium iodine (**red**). 100× magnification.

We next assessed the capacity of a fluorescent variant of CIGB-210 to penetrate in MT4 cells. MT4 cells were treated with 10, 20 or 40 µM of fluorescein labeled CIGB-210 for 15 min, 60 min or 24 h. The percentage of fluorescent cells, as determined by flow cytometry, increased with both the incubation time and peptide concentration (Figure 9A,B). The potential contribution of the peptides attached to the cell surface was excluded by introducing a quenching step with Trypan blue as have been reported elsewhere [29,30]. A biotinylated variant of CIGB-210 was detected by fluorescence microscopy in MT4 cells treated for 24 h (Figure 9E). CIGB-210 internalized the MT4 cells and exhibited a maximum penetration of 83% ± 3% fluorescent cells after 24 h of incubation at 40 µM.

### 3.9. Effect of CIGB-210 on Vimentin Expression in MT4 Cells

**Figure 10:**
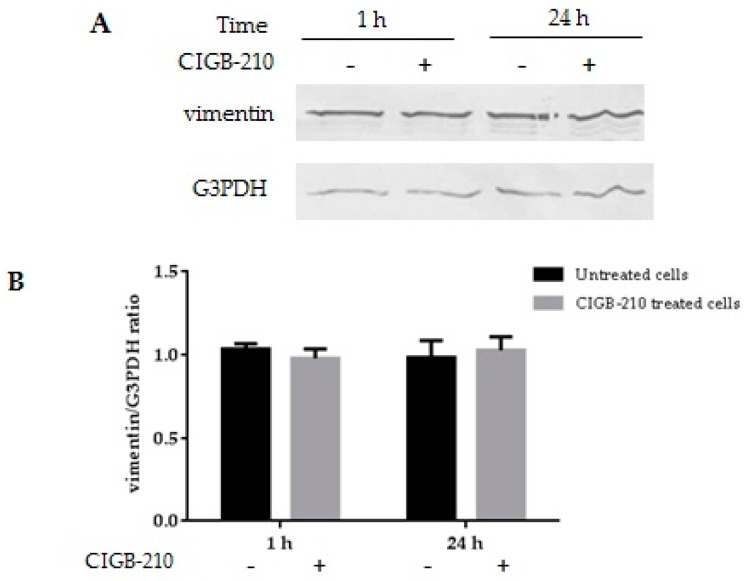
Effect of CIGB-210 on vimentin expression evaluated by Western blot. (**A**) MT4 cells were treated with CIGB-210 (200 µM) for 1 and 24 h. MT4 cells cultured in the absence of the peptide during the same time periods were used as controls. G3PDH served as a loading control; (**B**) Band intensities were quantified using Image J software and the ratio vimentin/G3PDH was calculated. Graphs represent averages of quantifications and error bars mean standard deviation. Mann–Whitney test was applied for calculation of significance; *p* > 0.05. Data are representative from three experiments.

MT4 cells were treated for 1 or 24 h with CIGB-210 and the levels of vimentin expression were evaluated by Western blot (Figure 10). G3PDH was detected as the normalizing protein. Similar intensities in the vimentin bands were observed in the samples from control MT4 cells and in CIGB-210 treated cells.

## 4. Discussion

The need for a more efficient therapy against HIV has led to a continuous search for new biological approaches to interfere with viral replication. One such strategy has been the use of cellular proteins as targets for therapy [10,32,33]; an approach endowed with the additional benefit that it may help curtail the emergence of resistant viral strains [17,34].

A proteomic analysis conducted on MT4 cells revealed that vimentin was down-regulated whenever the cells were treated with a DLE fraction that exhibited anti-HIV activity. This finding led us to explore the effect of a vimentin knockdown on the early stages of HIV-1 replication, using a single-round challenge system consisting of an eGFP-expressing, self-inactivating HIV-1 pseudotype (pLGW) that does not use HIV receptors for entry. The important reduction that we observed for the number of eGFP-positive cells in the vimentin knockdown MT4 line suggests that this protein does play a role in the early steps of HIV-1 replication, although the experimental system employed in this case does not enable dissecting the potential involvement of vimentin in later stages of the HIV replication cycle or virus entry.

A multi-round infectivity assay was next used to evaluate the effect of a vimentin knockdown, as this type of experimental setup has received extensive use for evaluating the activity of antiretroviral compounds [35,36]. This assay also revealed that HIV-1 replication was significantly reduced in MT4sh/Vim (a stable vimentin knockdown cell line), furthering the notion that vimentin plays an important role in HIV-1 replication.

Shoeman *et al.* [19] have reported that vimentin is a substrate for HIV-1 PR. Moreover, treatment with vimentin N-terminal peptides obtained by proteolytic processing triggered a rearrangement of the cell nucleus architecture that reminisces that observed in HIV-infected cells [23]. These findings raise the possibility that vimentin cleavage may play a role in the cytopathology of HIV infections.

It has also been reported that Vif, the viral infectivity factor involved in the processes of viral RNA folding and packaging [37], colocalizes with vimentin [25]. Further research would thus be required to determine the exact role of vimentin in HIV replication. As an IF forming protein, vimentin is most likely involved in the transportation of the integration complex to the nucleus, viral morphogenesis or budding.

A relationship between vimentin and different viruses, including HIV [23,38,39], has been previously documented [40,41,42,43]. However, none of these studies has reported the impact that a reduction of vimentin expression may have on viral replication. To our knowledge, this is the first publication showing that reducing the levels of cellular vimentin inhibits HIV-1 replication.

Transmission electron microscopy (TEM) in MT4 cells treated with the anti-HIV fraction B1 revealed modified IFs as compared to untreated MT4 cells. TEM showed apparently shorter filament than untreated cells, however, fluorescence microscopy revealed that change can be essentially described as the loosing of the polarized distribution pattern of vimentin and the reorganization of vimentin IFs in a network like structure. A similar modification of IFs was observed in the stable vimentin knockdown cell line and cells treated with CIGB-210. Although TEM alone cannot identify the specific protein forming the observed IFs, it seems highly plausible that these are IFs composed of vimentin, which is the major constituent of these structures in leukocytes [44]. Filament diameters were between 9 to 10 nm as correspond to IFs. In addition, the location of the IFs is coherent with the perinuclear localization of vimentin. Moreover, immunofluorescence microscopy images clearly identified the changes in vimentin IFs.

The reduction in the levels of cellular vimentin observed after treatment with the B1 fraction or upon siRNA knockdown may be influencing the architecture of the IFs. That premise led us to hypothesize that it would be possible to inhibit HIV-1 infection by modulating cellular vimentin IFs, either through a reduction in vimentin levels or by inducing structural changes in IFs. To validate this hypothesis, we used a peptide that has been reported to disassemble vimentin IFs *in vitro* and in fibroblast cell lines, probably by binding to homologous sequences in the alpha helixes that associate to form the IFs [31].

The effect of CIGB-210 on the cellular distribution of vimentin IFs was assessed by fluorescence microscopy. The treatment with CIGB-210 also changed the polarized distribution of vimentin IFs on MT4 cells by inducing a reorganization of the IF network throughout the cell cytoplasm forming a network around the cell nucleus.

The structural changes induced by peptide CIGB-210 on IFs were indeed similar to those observed in MT4sh/Vim cells or in MT4 cells treated with the B1 fraction. Furthermore, CIGB-210 exhibited a potent antiviral activity against HIV-1, confirming the hypothesis that it is possible to inhibit HIV-1 replication by acting on vimentin IFs. Interestingly, CIGB-210 did not change vimentin levels, indicating that a modification in the structure or cellular distribution of IFs was sufficient for inhibiting HIV-1 replication.

The up-take study of CIGB-210 showed that the peptide is capable of penetrating MT4 cells. CIGB-210 exhibited a slower kinetics of penetration as compared to a peptide including the sequence of Tat cell penetrating peptide. However, after 24 h of incubation, the time period we used to demonstrate anti-HIV antiviral activity, more than 80% of the cells had internalized the peptide.

The low levels of HIV replication in cells with reduced vimentin expression (MT4sh/vim), and the ability of a peptide that modifies vimentin IFs to inhibit HIV replication suggest that a reduction in vimentin levels or a change in the distribution of vimentin IFs, led to an efficient HIV inhibition in MT4 cells.

Taken together, our results suggest that vimentin can be a suitable target for inhibiting HIV-1. Since vimentin is a genetically conserved host factor, any drug targeting this protein would have a lower probability of selecting for drug-resistant viruses. CIGB-210 exhibited very low cytotoxicity and a potent, dose-dependent inhibitory activity on HIV-1 replication. Its high safety index makes this peptide an attractive drug candidate against HIV-1. Further studies will be required to fully understand the specific role of vimentin on HIV infection and to more precisely define the mechanism by which CIGB-210 inhibits HIV-1.

## Supplementary Materials

The following are available online at http://www.mdpi.com/1999-4915/8/6/98/s1, Figure S1: Diagram of the expression transfer plasmid encoding shRNA targeting vimentin. Figure S2: Viability of MT4 cells treated with the B1 fraction.
