# Supplementary material for: Identification of Vimentin as a Potential Therapeutic Target against HIV Infection
Source: Viruses. 2016 Jun 15;8(6):98. doi: 10.3390/v8060098 (PMC4926169; doi:10.3390/v8060098)
Supplement: Supplementary file 1 [file viruses-08-00098-s001.pdf]

Supplementary Materials: Identification of Vimentin as a Potential Therapeutic Target against HIV Infection

Celia Fernández-Ortega, Anna Ramírez, Dionne Casillas, Taimi Paneque, Raimundo Ubieta, Marta Dubed, Leonor Navea, Lila Castellanos-Serra, Carlos Duarte, Viviana Falcon, Osvaldo Reyes, Hilda Garay, Eladio Silva, Enrique Noa, Yassel Ramos, Vladimir Besada and Lázaro Betancourt

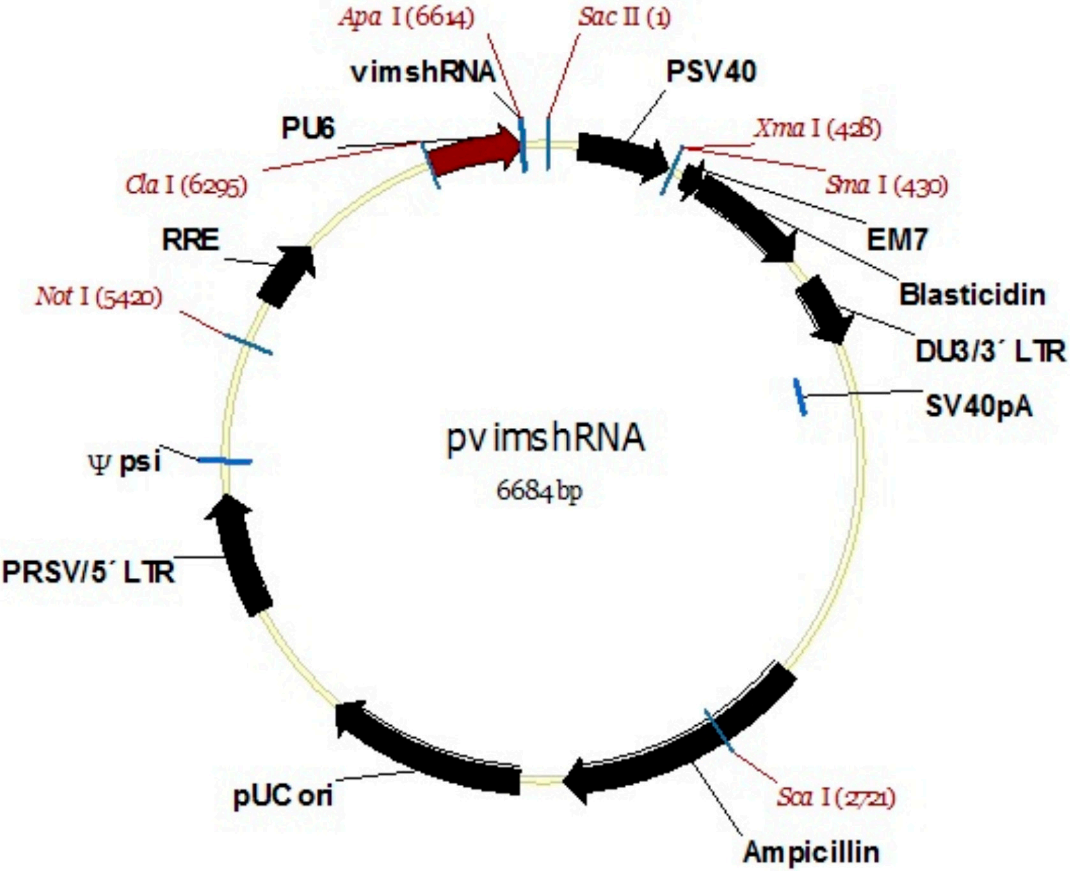

Figure S1. Diagram of the expression transfer plasmid encoding shRNA targeting vimentin. The plasmid contains RSV enhancer-promoter and HIV-1 5′ LTR (P_{RSV}/5′ LTR), HIV-1 packaging signal (Ψ psi), HIV-1 Rev response element (RRE), U6 promoter (PU6), short hairpin RNA targeting vimentin (vim shRNA), SV40 early promoter and origin (P_{SV40}), Blasticidin resistance gene and ΔU3/HIV-1 truncated 3′ LTR (ΔU3/3′ LTR).

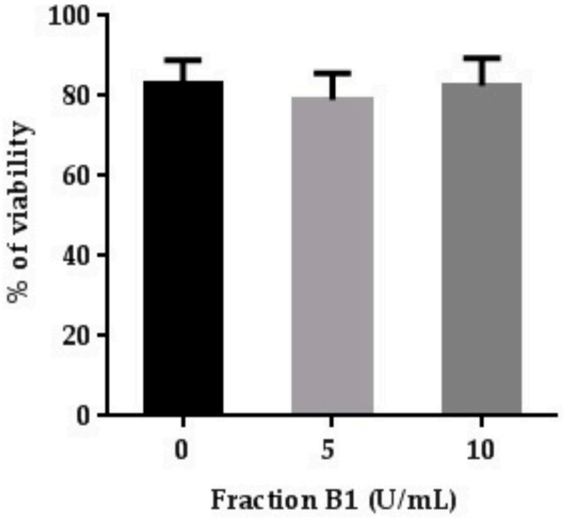

Figure S2. Viability of MT4 cells treated with the B1 fraction. MT4 cells were treated with either 5 U/mL or 10 U/mL of B1 fraction for 192 h at 37 °C in 5% CO_2. The trypan blue assay was performed to determine the cell viability. One representative experiment out of three is shown. Bars indicate the mean and the standard deviation of the samples run in triplicate. Kruskal–Wallis test was applied for calculation of significance among groups; $p > 0.05$. The treatment with the B1 fraction showed no cytotoxic effect in MT4 cells.
